# Identification of Mesencephalic Astrocyte-Derived Neurotrophic Factor as a Novel Neuroprotective Factor for Retinal Ganglion Cells

**DOI:** 10.3389/fnmol.2017.00076

**Published:** 2017-03-17

**Authors:** Feng-Juan Gao, Ji-Hong Wu, Ting-Ting Li, Shan-Shan Du, Qiang Wu

**Affiliations:** ^1^Department of Ophthalmology, Shanghai Jiao Tong University Affiliated Sixth People’s HospitalShanghai, China; ^2^Eye and ENT Hospital, State Key Laboratory of Medical Neurobiology, Institutes of Brain Science and Collaborative Innovation Center for Brain Science, Shanghai Medical College, Fudan UniversityShanghai, China; ^3^Shanghai Key Laboratory of Visual Impairment and RestorationShanghai, China; ^4^Key Laboratory of Myopia, Ministry of Health, Fudan UniversityShanghai, China; ^5^Department of Ophthalmology, The First Affiliated Hospital of Zhengzhou UniversityZhengzhou, China; ^6^Shanghai Key Laboratory of Diabetes Mellitus, Shanghai Jiao Tong University Affiliated Sixth People’s HospitalShanghai, China

**Keywords:** mesencephalic astrocyte-derived neurotrophic factor, retinal ganglion cells, neuroprotective, endoplasmic reticulum stress, retinal pathology

## Abstract

Mesencephalic astrocyte-derived neurotrophic factor (MANF), a newly discovered secreted neurotrophic factor, has been proven to not only protect dopaminergic neurons and other cell types but also regulate neuroinflammation and the immune response to promote tissue repair and regeneration. However, to date, there is no information regarding the relationship between MANF and retinal ganglion cells (RGCs) in the eye. In the current study, we first determined the expression of MANF in the retina and vitreous. Then, we examined the effect of MANF on RGCs using both *in vivo* and *in vitro* models and simultaneously explored the underlying neuroprotective mechanisms of MANF. Finally, we measured the concentrations of MANF in the vitreous of patients with different retinopathies. We demonstrated that MANF was highly expressed in RGCs and that exogenous MANF could protect RGCs from hypoxia-induced cell injury and apoptosis both *in vitro* and *in vivo* by preventing endoplasmic reticulum stress-mediated apoptosis. Furthermore, MANF can be detected in the vitreous humor, and the concentration changed under pathological conditions. Our results provide important evidence that MANF may be a potential therapeutic protein for a range of retinal pathologies in either the preclinical stage or after diagnosis to promote the survival of RGCs. Vitreous MANF may be a promising protein biomarker for the indirect assessment of retinal disorders, which could provide indirect evidence of retinal pathology.

## Introduction

Mesencephalic astrocyte-derived neurotrophic factor (MANF), also known as Armet, was first isolated from a rat type-1 astrocyte ventral mesencephalic cell line. MANF is widely expressed in the nervous system and non-neuronal tissue, such as the cerebral cortex, liver, and heart (Lindholm et al., 2008). Because MANF is a secreted protein, its expression is especially abundant in secretory cells and tissues, including human serum, salivary glands, and embryonic pancreas. Recently, studies conducted at the University of Helsinki demonstrated that the concentration of MANF is increased in the serum in children with type 1 diabetes, which indicated that MANF may be used as a potential therapeutic protein for diabetes (Galli et al., 2016). Furthermore, our recent studies showed that MANF could be robustly expressed in RGCs and that hypoxia can stimulate the up-regulation of MANF expression over time, which suggested that MANF may play a vital role in the functional regulation of RGCs in both health and disease (Gao et al., 2017).

Recent advances have indicated that MANF could protect dopaminergic neurons and cell types in animal models of Parkinson’s disease, Alzheimer’s disease, and brain ataxia (Petrova et al., 2003; Lindholm et al., 2007). MANF is a new secretory neurotrophic factor, and its expression and secretion are regulated by endoplasmic reticulum stress (ERS) (Apostolou et al., 2008; Tadimalla et al., 2008). MANF not only modifies ER homeostasis to protect cells against ERS-induced cell death but also regulates neuroinflammation and the immune response to promote tissue repair and regeneration (Neves et al., 2016; Zhu et al., 2016). The mechanisms of the neuroprotective effects of MANF in rodent models of cerebral ischemia and spinocerebellar ataxia are mediated by inhibition of ischemia-induced apoptosis (Yang et al., 2014). Numerous eye diseases are associated with neuronal apoptosis, ERS, inflammation, and ischemia, such as diabetic retinopathy (Ikesugi et al., 2006; Li et al., 2009; Tang and Kern, 2011; Zhang et al., 2015), glaucomatous optic neuropathy (Yang et al., 2011; Ha et al., 2015), retinal detachment (Liu et al., 2010; Zhu et al., 2013), and age-related macular degeneration (Li et al., 2008; Libby and Gould, 2010). These findings suggest that MANF may be a promising therapeutic strategy for promoting the survival of retinal neurons. Therefore, we hypothesized that MANF may have therapeutic potential for the treatment of retinopathy. However, there is little research available on this subject. The only study on MANF in the retina was reported in *Science* and demonstrated for the first time that MANF is expressed in the mouse retina and exerts immune modulatory function to promote tissue repair and successful regeneration in the retina (Neves et al., 2016). These results provided a strong foundation for our current study and revealed that further attention and investigation of the expression, function, and effect of MANF in retinopathy are imperative.

In the current study, we first determined the expression of MANF in the retina and vitreous. Then, we assessed the effect of MANF on retinal ganglion cells (RGCs) using both *in vivo* and *in vitro* models and simultaneously explored the underlying neuroprotective mechanisms. Finally, we measured the concentrations of MANF in the vitreous of patients with different retinopathies. We demonstrated that MANF was highly expressed in the RGCs and that exogenous MANF could protect RGCs from hypoxia-induced cell injury and apoptosis both *in vitro* and *in vivo* by preventing ERS-mediated apoptosis. MANF can be detected in the vitreous humor, and the concentration of MANF changed under pathological conditions.

## Materials and Methods

### Animals and Ethics Statement

A total of 92 male Sprague-Dawley rats (150–200 g) and 150 newborn Sprague-Dawley rats (1–4 days) (SLAC Laboratory Animal Co., Ltd. Shanghai, China) were used. This study was carried out in accordance with the guidelines of Shanghai Jiaotong University Affiliated Sixth People’s on the ethical use of animals. The protocol was reviewed and approved by the animal experimental ethics committee of Shanghai Jiaotong University Affiliated Sixth People’s. The animal handling and experimental protocols were carried out in accordance with the approved guidelines of Animal Care and Use Committee of Research Ethical Committee, Shanghai Jiaotong University Affiliated Sixth People’s and the Association Research in Vision and Ophthalmology (ARVO) Statement for the Use of Animals in Ophthalmic and Vision Research. Animals were kept in standard cages at room temperature (15–25°C) and 12-h light/12-h dark schedule with food and water available *ad libitum*.

Sprague-Dawley rats were allocated randomly into normal control (NC) group (*n* = 26), chronic ocular hypertension (COHT) group (*n* = 32), COHT + PBS group (*n* = 8, intravitreous injection of 2 μl PBS) and COHT + MANF group (*n* = 26, intravitreous injection of 2 μl, 1 μg/ml). In this study, all animals were anesthetized by intraperitoneal injection of 10% chloral hydrate.

### Patients and Vitreous Samples and Ethics Statement

In this study, vitreous samples were obtained from 55 patients undergoing vitreoretinal surgery, including 21 proliferative diabetic retinopathy, 16 retinal detachment and 18 macular hole patients. The mean age was 48.71 ± 11.83 years (range 29–65 years). This study was carried out in accordance with the recommendations of Shanghai Jiaotong University Affiliated Sixth People’s Hospital with written informed consent from all subjects. All subjects gave written informed consent in accordance with the Declaration of Helsinki. The protocol was approved by the Office of Research Ethical Committee, Shanghai Jiaotong University Affiliated Sixth People’s Hospital. Undiluted vitreous samples were obtained at the onset of vitrectomy and frozen at -80°C immediately until assayed. Healthy vitreous was obtained from the Shanghai Jiaotong University Affiliated Sixth People’s Hospital following the guidelines of the Shanghai Jiaotong University Clinical Human Research Ethics Committee (*n* = 2). Both were from donors who had been screened to make sure there was no underlying ocular disease.

### Cell Culture and Treatment

Primary cultured RGCs were purified by two-step immunopanning method as we described previously (Gao et al., 2016). RGCs were collected and seeded into 96, 24 and 6-well plates pretreated with mouse-laminin (Trevigen Inc., Gaithersburg, MD, USA) and poly-D-lysine (Sigma–Aldrich, St. Louis, MO, USA). Plates were incubated in a humidified incubator with 5% CO_2_ at 37° C. RGCs purity was checked by Thy1.1 and Brn 3b immunocytochemical staining (about 85%) (Gao et al., 2016). Forty-eight hours after seeding, RGCs were incubated with 200 μM cobalt chloride (CoCl_2_, Sigma–Aldrich, St. Louis, MO, USA) to induce hypoxia and apoptosis (Kim et al., 2013), combined with 0, 10, 20, 50, or 100 ng/ml MANF for 24 h, then incubated 12 h, 24 and 48 h under optimal concentration. Half of the RGC medium was replaced with fresh RGC medium every 3 days.

### Intraocular Pressure (IOP) Elevation and MANF Treatment

Elevation of IOP was induced by injection of 5 μl of paramagnetic polystyrene microbeads (FluoSpheres; Invitrogen, Carlsbad, CA, USA; 15-μm diameter) into the anterior chamber of the right eye of each animal under a surgical microscope, as previously reported (Sappington et al., 2010). IOP was measured every 3 days after the injection using a rebound tonometer (Icare^®^ Tonolab, TioLat, Helsinki, Finland). All the measurements were performed between 9 and 10 am by the same operator, and the IOP is reported as the mean ± standard deviation (SD) of the average of three independent readings.

A dose of 2 μl of MANF (1 μg/ml, PeproTech, Rocky Hill, NJ, USA) was intravitreally injected 2 days before increased IOP was induced and then once a week for 2 weeks.

### Immunofluorescence

Immunofluorescence staining was conducted as previously described Wu et al. (2013). The eyes and RGCs from different groups were fixed with 4% paraformaldehyde (PFA) for 2 h and 20 min at room temperature, respectively. Then, the eyes were cut into 8-μm-thick sections after graded dehydration with 20 and 30% sucrose solution and then stored at -80°C until use. Sections and RGCs were incubated in 0.1% Triton X-100 and 3% (w/v) bovine serum albumin (BSA) (Sigma–Aldrich, St. Louis, MO, USA) for 30 min sequentially at room temperature to prevent non-specific background. Then, sections and RGCs were incubated with rabbit anti-MANF (1:200, Abcam, Cambridge, MA, USA) and anti-survivin (1:200, Abcam, Cambridge, MA, USA) antibody at 4°C overnight, followed by successive incubations with fluorescein-conjugated goat anti-rabbit secondary antibody (1:400, Molecular Probes, USA) and Hoechst staining. Negative controls were routinely prepared by incubating the cells and retinal sections in normal buffered serum instead of the primary antibody. The stained sections and cells were visualized and imaged using confocal microscopy (Leica SP8, Hamburg, Germany).

### Cell Counting Kit-8 (CCK8) Assay for RGC Viability

CCK8 solution (Dojindo Laboratories, Kumamoto, Japan) was added to 96-well plates (10 μl/well). After a 4-h incubation at 37°C, the plates were analyzed at 450 nm with the Tecan Genios microplate reader (Synergy H1, BioTAK). All values are expressed as the mean ± SD of at least three wells and at least three separate experiments. As the initial cell density was the same, higher OD values represent increased cell viability over time. We compared the changes in the OD values at different times to evaluate the RGC viability.

### Caspase 3/7 Activity

Approximately 10,000 purified RGCs were seeded into 96-well plates. The caspase 3/7 activation in the different groups was evaluated by the Caspase-Glo 3/7 assay (Promega, Milano, Italy), as previously reported Park et al. (2016). The luminescence signals from the cell lysate solution were determined with the Tecan Genios microplate reader (Synergy H1, BioTAK). All experiments were performed in triplicate.

### Flow Cytometry Analysis of Apoptosis

The proportion of apoptotic cells was measured using a FACSCalibur platform according to the manufacturer’s instructions for the Annexin V-FITC/PI flow cytometric assay kit (Becton Dickinson, San Jose, CA, USA). Briefly, after each treatment, the cells were collected, washed, and resuspended in phosphate-buffered saline (PBS) containing Annexin V-FITC and PI for 20 min in the dark at 37°C in 5% CO_2_. Then, the stained cells were analyzed using a FACSCalibur platform. The percentage of apoptotic cells was determined by calculating the number of annexin V+/PI-cells. All experiments were performed in triplicate.

### Western Blot Analysis

To obtain a high protein concentration from the vitreous samples for Western blot analysis, protein precipitation must be the first step. In the current study, the vitreous was concentrated using the acetone method as described by Jiang et al. (2004). Primary cultured RGCs and retinal protein extractions were performed as previously described Wu et al. (2015). Then, the protein concentration was quantified using a BCA protein assay kit (Thermo Fisher Scientific, Rockford, IL, USA); the proteins were separated by SDS-polyacrylamide gel electrophoresis; and the separated proteins were electrotransferred to polyvinylidene difluoride membranes. After blocking with 5% non-fat milk for 30 min, the membranes were incubated overnight at 4°C with primary antibodies against MANF, survivin, ERS-associated protein CCAAT-enhancer-binding protein (C/EBP), homologous protein (CHOP), cleaved caspase-3 antibody, and β-actin (Abcam, Cambridge, MA, USA). The secondary antibodies included HRP-conjugated goat anti-rabbit antibody (Millipore, Billerica, MA, USA) and HRP-conjugated goat anti-mouse antibody (Millipore, Billerica, MA, USA). The Western blots were imaged with the Kodak Imaging System (Kodak 440CF) using the ECL Western blotting substrate (Hyperfilm ECL, Thermo Fisher Scientific, Rockford, IL, USA). The intensity of the band was quantified by densitometry using ImageJ software (NIH, Bethesda, MD, USA).

### TUNEL Assay

TUNEL staining was performed according to the manufacturer’s instructions (In Situ Cell Detection Kit; Roche, Mannheim, Germany) as we previously reported Fu et al. (2016). Cultured RGCs were fixed in 4% (w/v) PFA at 4°C for 30 min and then blocked with 3% BSA and 0.1% Triton X-100 for 1 h at room temperature. The TUNEL reaction mixture was added to the samples and incubated for 60 min at 37°C in the dark. After 3 rinses with PBS, the samples were stained with DAPI (1:2000; Life Technologies, Molecular Probes, NucBlue Fixed Cell Stain, Eugene OR, USA). The TUNEL-positive cell nuclei were visualized using a confocal microscope (Leica SP8, Hamburg, Germany) and quantified using ImageJ software. Six microscope fields of view from six different wells were analyzed per treatment condition.

### Photopic Negative Response (PhNR) Recordings

The PhNR was recorded to evaluate RGC function at baseline and after 3, 7, and 14 days of IOP elevation as previously reported Rangaswamy et al. (2007) and Porciatti (2015). After the rats were anesthetized, the pupils were dilated with phenylephrine hydrochloride and tropicamide, and two 3-mm platinum wire loop electrodes were then placed on the corneal surface of the eyes. One subdermal needle electrode inserted at the base of the right leg served as the ground, and the other subdermal needle electrode placed over the nasal bone acted as the common reference. Light stimuli were delivered using a ColorDome unit. Four different stimuli of 11.38 cd.s/m^2^-0.33 Hz, 11.38 cd.s/m^2^-1 Hz, 22.76 cd.s/m^2^-0.33 Hz, and 22.76 cd.s/m^2^-0.33 Hz were used in a four-step examination. In each step, the stimulus frequency was 2 Hz, with a 4-ms exposure to a green light on a green background with an intensity of 10 cd/m^2^ displayed by the Espion Visual Electrophysiology System (Espion E3, Diagnosys, Diagnosys UK Ltd, UK). The PhNR amplitude was recorded from the baseline to the trough of the negative response.

### Retrograde Labeling and Counting of RGCs

The procedures were performed as previously reported (Wu et al., 2013). Briefly, Fluoro-Gold (FG, Sigma–Aldrich, St. Louis, MO, USA) was injected into the superior colliculus on each side after the rats in each of the four groups were deeply anesthetized. One week post-injection, the rat eyes were harvested and placed in 4% PFA for 2 h at room temperature, and the whole retina was then carefully dissected and flat-mounted on a slide. The RGCs were counted and averaged per eight microscopic fields of identical size at a distance of 1–2 mm from the optic nerve head where the RGC densities were comparable at 200 × magnification. Each group contained at least three rats for measurement of the mean density. The RGCs were manually counted by two operators in a blinded manner using ImageJ software (NIH, Bethesda, MD, USA). The density of RGCs is expressed as the number of cells per mm^2^.

### Enzyme-Linked Immunosorbent Assay (ELISA)

The levels of vitreous MANF expression were measured by human ELISA kits (CUSBIO Life Science, College Park, MD, USA) according to the manufacturer’s instructions. The vitreous samples were diluted 10 times before analysis. The acceptance criteria of the assay followed the previously published recommendations (DeSilva et al., 2003). The MANF concentration was calculated from the standard curve of six individual assays.

### Statistical Analysis

All data are expressed as the means ± SD and were analyzed using SigmaStat software. All data were assessed by the Shapiro–Wilk test, and only variables that were normally distributed were tested; we also analyzed the variance of the data, followed by performance of the two-tailed unpaired Student’s test. Values of *p* < 0.05 were considered the threshold for statistical significance.

## Results

### Expression and Distribution of MANF in the Normal Rat Retina

**FIGURE 1:**
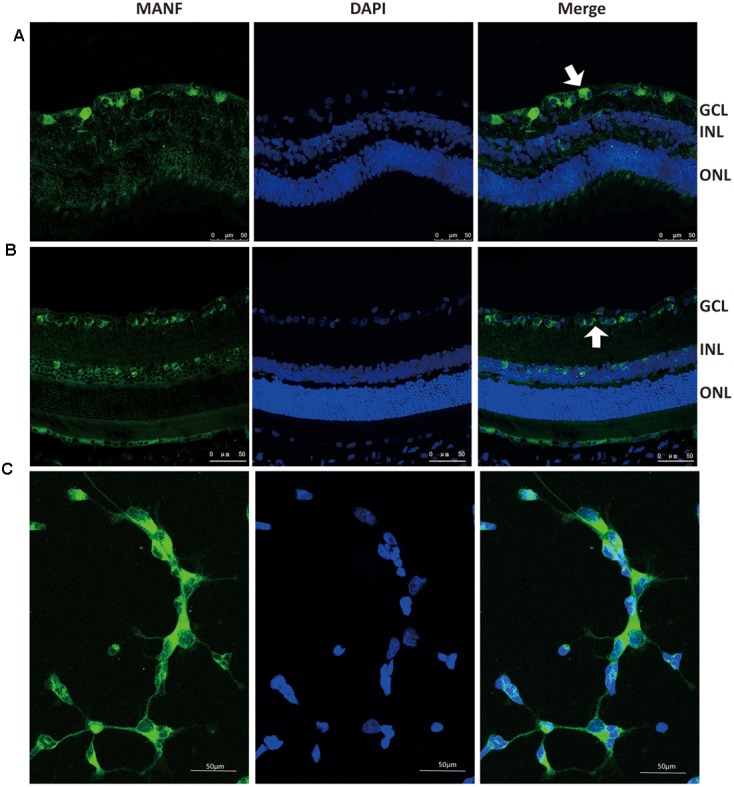
**Immunofluorescence staining of MANF protein in rat cultured RGCs (C)**, human retina **(A)**, and Sprague-Dawley rat retina **(B)**. MANF staining was mainly distributed throughout the GCL, with some labeling in the INL (indicated by arrowheads). The nuclei were labeled with DAPI (blue). Magnification 400X; Scale bar: 50 μm. ONL, outer nuclear layer; INL, inner nuclear layer; GCL, ganglion cell layer.

As we have previously described Gao et al. (2017), immunofluorescence staining shows that MANF is predominantly localized in the ganglion cell layer and inner nuclear layer in both rat and human retinas (**Figures 1A,B**). To determine if the MANF expression in the ganglion cell layer was specific to the RGCs, primary cultured RGCs were used for *in vitro* validation. The results confirmed that MANF was robustly expressed in the RGC somas (**Figure 1C**). These observations suggest that MANF may play a potential role in the pathophysiology of the retina, especially of the RGCs.

### MANF Prevents Hypoxia-Induced RGC Apoptosis

**FIGURE 2:**
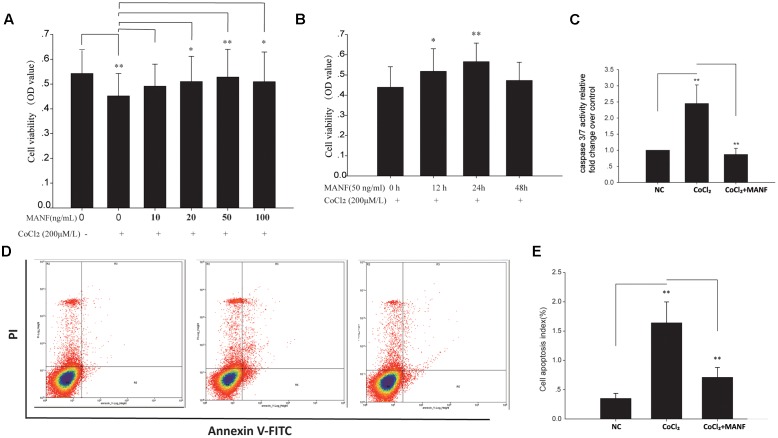
**(A)** The viability of RGCs treated with 0–100 ng/ml MANF with or without CoCl_2_ was measured by the CCK-8 assay. The bars represent the means ± SD, ^∗^*p* < 0.05, ^∗∗^*p* < 0.01. **(B)** The viability of RGCs treated with 50 ng/ml MANF and 200 μM CoCl_2_ for 0, 12, 24, and 48 h. The bars represent the means ± SD. ^∗^*p* < 0.05, ^∗∗^*p* < 0.01. **(C)** Caspase 3/7 activities were measured by a luciferase assay. The bars represent the means ± SD. ^∗∗^*p* < 0.01. **(D)** Apoptosis was detected by Annexin V and propidium iodide (PI) staining in RGCs treated with 50 ng/ml MANF and 200 μM CoCl_2_ for 24 h. The R4 quadrant (Annexin V-/PI-), R5 quadrant (Annexin V+/PI-), and R3 quadrant (Annexin V+/PI+) indicate the percentage of viable cells, apoptotic cells, and necrotic cells, respectively. **(E)** The percentage of apoptotic cells following CoCl_2_ treatment with or without MANF. The values represent the mean ± SD of three independent experiments. ^∗∗^*p* < 0.01.

To determine whether MANF exerts neuroprotective effects on the RGCs, primary RGCs were cultured under hypoxia with or without exogenous MANF. We found that CoCl_2_ significantly inhibited cell viability (OD value:0.548 ± 0.086 vs. 0.438 ± 0.078, *n* = 9, *p* < 0.01, **Figures 2A,B**), while MANF rescued cell viability in a dose- and time-dependent manner in the RGCs treated with CoCl_2_. Because cell viability reached the optimum level at 50 ng/ml for 24 h (OD value:0.576 ± 0.069, *p* = 0.0081, <0.01, **Figures 2A,B**), we used 50 ng/ml as the optimal effective concentration in subsequent studies.

**FIGURE 3:**
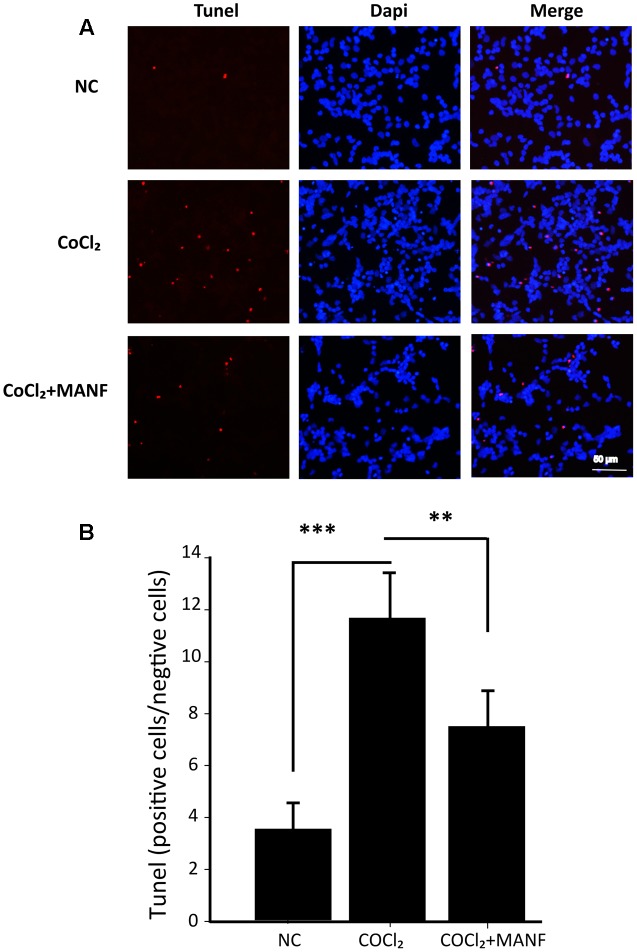
**(A)** TUNEL analysis of RGCs from three different groups: NC group, CoCl_2_ treatment group (24 h), and CoCl2 with MANF treatment group (24 h). Red indicates TUNEL-positive cells, and blue indicates DAPI. All images were captured at the same magnification. Scale bar, 50 μm. **(B)** Quantitative analysis of TUNEL-positive cells in the different treatment groups. The data are presented as the means ± SD. ^∗∗∗^*p* < 0.001, the hypoxia group compared with the NC group; ^∗∗^*p* < 0.01, the hypoxia + MANF group compared with the hypoxia group.

Furthermore, the protective effect of MANF was evaluated by measuring the caspase 3/7 activity using a luminescent caspase activity assay kit (**Figure 2C**). Caspase 3/7 activity was significantly increased under hypoxia (1.45 ± 0.48-fold, *p* = 0.0069, <0.01), whereas MANF treatment remarkably decreased caspase 3/7 activity by 0.65 ± 0.16-fold compared with the hypoxia group (*p* = 0.0076, <0.01). These results suggested that MANF could inhibit CoCl_2_-induced caspase 3/7 activation. RGC apoptosis and death were also evaluated by the flow cytometry-based Annexin V + PI assay and TUNEL staining. As shown in **Figures 2D,E**, **3**, the numbers of annexin V (+) and TUNEL-positive RGCs were significantly increased under hypoxia (4.69 ± 0.78-fold, *p* < 0.01 vs. 5.98 ± 1.05-fold, *p* < 0.001), whereas MANF treatment remarkably decreased the number of apoptotic RGCs (decreased by 0.57 ± 0.12-fold, *p* < 0.01 vs. 0.48 ± 0.11-fold, *p* < 0.01). These results collectively demonstrated that MANF can protect RGCs from hypoxia-induced apoptosis *in vitro*.

### Mechanisms of the Protective Effect of MANF on RGCs

**FIGURE 4:**
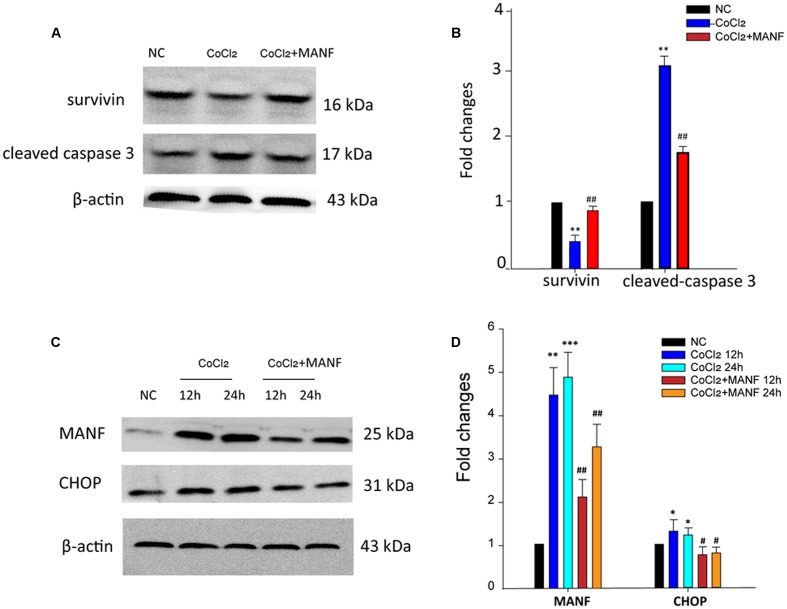
**Western blot analysis was performed**. The expression levels of cleaved caspase-3, survivin, MANF, and CHOP were determined in the RGCs from the three different treatment groups: NC group, CoCl_2_ treatment group, and CoCl_2_ with MANF treatment group. The bars represent the means ± SD. β-actin was used as an internal control. **(A)** and **(C)** Bands showing the protein expression. **(B)** and **(D)** Band density is represented as normalized ratios (β-actin used as control = 1). ^∗^*p* < 0.05, ^∗∗^*p* < 0.01, ^∗∗∗^*p* < 0.001, the hypoxia group compared with the NC group. #*p* < 0.05, ##*p* < 0.01, the hypoxia + MANF group compared with the hypoxia group.

For a further in-depth exploration of the mechanisms of the protective effect of MANF on RGCs under hypoxia, the levels of the anti-apoptotic survivin, apoptosis-related cleaved caspase-3, CHOP, and MANF proteins were analyzed by Western blotting (**Figure 4**). The results demonstrated that the expression levels of survivin were reduced (*p* = 0.0092, <0.01), while the levels of cleaved caspase-3, the activated form of caspase-3, were significantly upregulated (*p* = 0.0077, <0.01) after 24 h of CoCl_2_ treatment (**Figures 4A,B**). However, these changes were remarkably reversed by MANF treatment (*p* = 0.0056, <0.01). CHOP, a pro-apoptotic factor, was increased by >1.39 ± 0.12-fold and 1.49 ± 0.22-fold after 12 and 24 h of CoCl_2_ treatment, respectively, compared with the NC group, whereas MANF treatment markedly inhibited CHOP expression. Meanwhile, the expression levels of MANF were changed in a similar manner to CHOP (**Figures 4C,D**). These data indicated that MANF could protect against hypoxia-induced RGC apoptosis by upregulating the expression levels of survivin and suppressing the levels of cleaved caspase-3 and CHOP.

### Induction of Experimental Glaucoma

**FIGURE 5:**
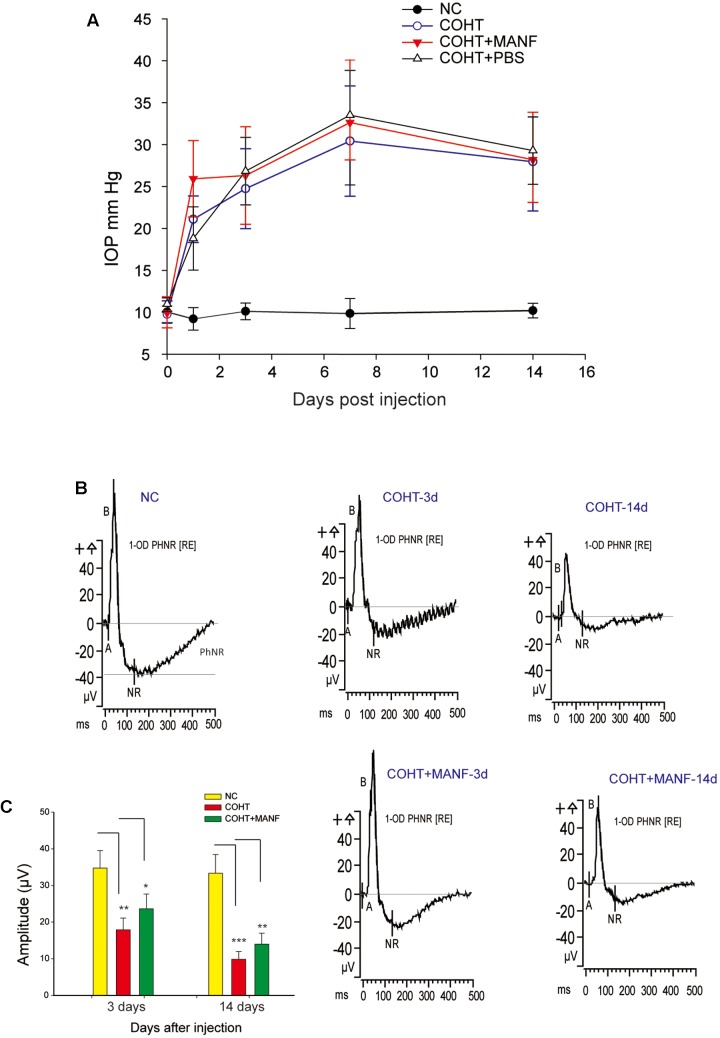
**(A)** The mean IOPs of the rats in the NC group (*n* = 22), COHT group (*n* = 28), COHT + PBS group (*n* = 8), and COHT + MANF group (*n* = 22). IOP was measured from 0 to 2 weeks after the microbead injections. The data are presented as the means ± standard deviations (SD). **(B)** The PhNR amplitudes at baseline (*n* = 22, 28, and 22), 3 days (*n* = 14, 25, and 22), and 2 weeks (*n* = 9, 16, and 15) in the NC group, COHT with MANF group, and COHT without MANF group, respectively. **(C)** Quantification of the mean amplitudes of the three groups. The data are expressed as the means ± SD. ^∗^*p* < 0.05, ^∗∗^*p* < 0.01, ^∗∗∗^*p* < 0.001.

To validate the protective effects of MANF on the RGCs, *in vivo* studies are needed. In the current study, a rat COHT model was used to establish the hypoxic injury to the RGCs. As shown in **Figure 5A**, significant IOP elevation (defined as 5 mmHg greater than the contralateral eye) was observed on the first day after the microbead injection (21.96 ± 3.64 mmHg vs. 9.82 ± 0.41 mmHg, *p* = 0.0016, <0.01), and the IOP remained elevated for an additional period of 2 weeks at significantly higher levels than those in the NC group at every time point over the entire 2-week study period (*t*-test, *p* = 0.028, <0.05 for each time point). MANF administration did not alter the IOP (*n* = 22, *p* = 0.32, >0.05). Therefore, the results demonstrated that the microbead injection resulted in COHT.

### MANF Rescues RGC Function *In vivo*

The function of the RGCs is impaired before death. To assess the functional changes in the RGCs, PhNR analysis was carried out (**Figures 5B,C**). The results showed that the PhNR amplitude was significantly reduced by approximately 48.52% after 3 days of COHT (*n* = 25, *p* = 0.0039, <0.01), which indicated that RGC function was significantly impaired in the early stage of COHT. With sustained COHT over time, the amplitudes of the PhNR declined further and were reduced by 69.43 ± 7.23% in the second week after COHT induction (*n* = 16, *p* = 0.0006, <0.001). As expected, MANF administration significantly rescued the amplitudes of the PhNR in the early stage of COHT, which had recovered by approximately 32.56 ± 5.25% on the third day (*n* = 22, *p* = 0.031, <0.05) and by 40.78 ± 7.11% in the second week (*n* = 15, *p* = 0.008, <0.01). Collectively, these results demonstrated that MANF effectively attenuated RGC functional impairment in the early stage of COHT, and the protective effect in the early stage of COHT was greater than that in the later stage of COHT (*p* < 0.05).

### MANF Promotes RGC Survival *In vivo*

**FIGURE 6:**
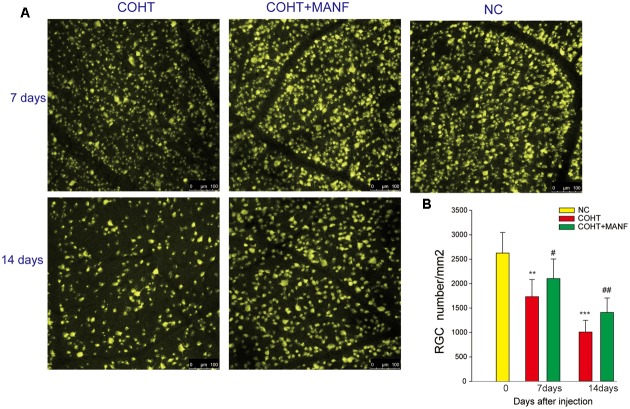
**(A)** Representative regions of FG-labeled RGCs in the flat-mounted retinas in the NC group and COHT with or without MANF groups after 0 (*n* = 4), 7 (*n* = 5, 8, and 7, respectively), and 14 (*n* = 5, 8, and 7, respectively) days after the microbead injections, and all images were captured at the same magnification. Scale bar, 100 μm. **(B)** Quantitation of the FG-labeled RGCs. The data are presented as the means ± SD, ^∗∗^*p* < 0.01, ^∗∗∗^*p* < 0.001, the COHT group compared with the NC group. #*p* < 0.05, ##*p* < 0.01, the COHT + MANF group compared with the COHT group at the same time point after the microbead injections.

To assess whether MANF administration could increase RGC survival under COHT, RGC loss was assessed by retrograde FG labeling (**Figure 6**). The mean RGC density in the eyes before microbead injection was 2633 ± 422 cells/mm^2^, and microbead-induced IOP elevation led to a gradual reduction of RGC somas over time (no evident differences during the first 3 days, *n* = 4, *p* > 0.01). The number of RGCs decreased by 33.98 ± 5.77% during the 1st week (*n* = 8, *p* = 0.0066, <0.01) and by 61.33 ± 8.21% during the 2nd week (*n* = 8, *p* = 0.0005, <0.001), while the number of RGCs increased by 20.33 ± 4.55% in the 1st week (*n* = 7, *p* = 0.041, <0.05) and by 39.77 ± 6.59% in the 2nd week (*n* = 7, *p* = 0.0078, <0.01) in the rats treated with MANF after COHT. These results indicated that MANF reduced the death of the RGCs after COHT and increased the survival rate of the RGCs.

**FIGURE 7:**
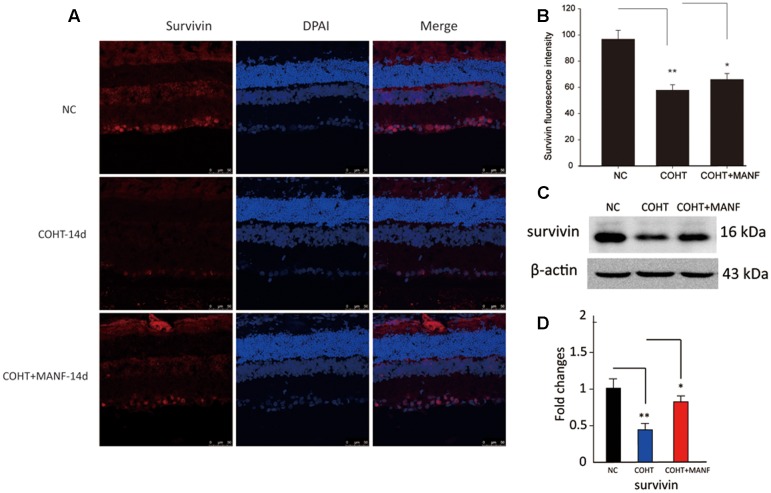
**(A)** Representative immunohistochemical images of survivin staining (red) in retinas from the three different treatment groups at 2 weeks after the microbead injections. The NC group (top, *n* = 8), COHT group (middle, *n* = 8), and COHT + MANF group (bottom, *n* = 8). Red indicates survivin staining; blue indicates DAPI. Scale bar, 50 μm. **(B)** Quantitative analysis of survivin-positive regions in the retinas from the different treatment groups. **(C,D)** Western blot analysis of survivin expression in the three different groups. β-actin was used as an internal control. The level of protein in each group is expressed as the value relative to the NC group. The data are presented as the means ± SD, ^∗^*p* < 0.05, ^∗∗^*p* < 0.01.

To further confirm the protective effect of MANF on RGCs under COHT, the levels of survivin, a protein that inhibits apoptosis, were measured by immunofluorescence staining and Western blot analysis (**Figure 7**). Quantitative estimation showed that the expression levels of survivin in the ganglion cell layer (GCL) and inner nuclear layer (INL) were significantly decreased by 0.42 ± 0.09-fold (immunofluorescence analysis, *n* = 6, *p* = 0.0042, <0.01) vs. 0.59 ± 0.17-fold (Western blot analysis, *n* = 6, *p* = 0.0076, <0.01) after 2 weeks of COHT. However, the survivin expression levels were markedly increased by 0.89 ± 0.21-fold (Western blot analysis, *n* = 6, *p* = 0.018, <0.05) in the retinas treated with MANF. Together, these findings strongly suggest that intravitreal delivery of MANF can promote the survival of RGCs in this model of chronic glaucoma.

### MANF in the Vitreous

**FIGURE 8:**
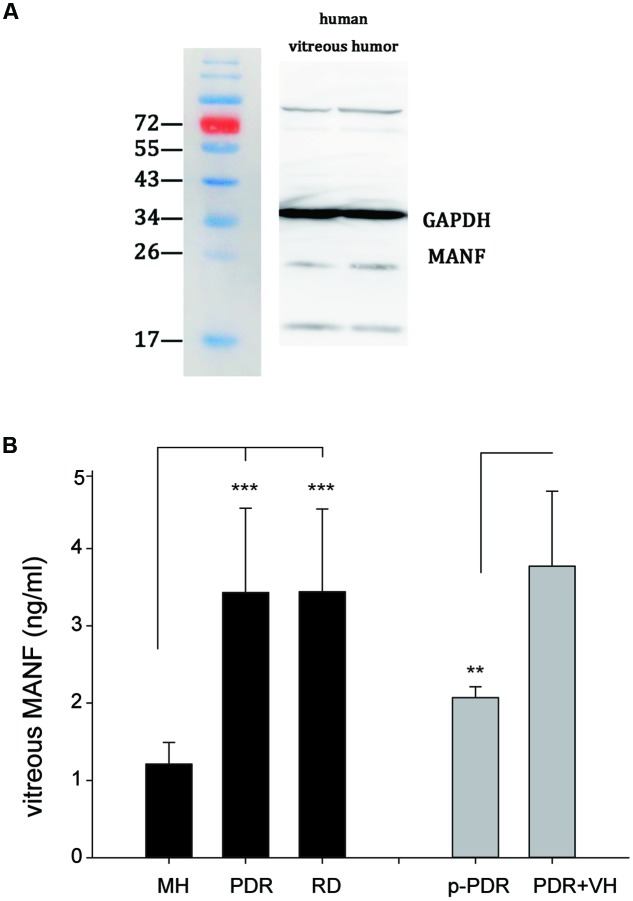
**(A)** Western blot analysis of MANF expression in the vitreous of human donors; a major band at the expected molecular weight (25 kDa) is apparent. **(B)** MANF concentrations in the vitreous of patients with MH (*n* = 18), PDR (*n* = 21), and RD (*n* = 16). The data are presented as the means ± SD, ^∗∗^*p* < 0.01, ^∗∗∗^*p* < 0.001. RD, retinal detachment; MH, macular hole; PDR, proliferative diabetic retinopathy; p-PDR, pure proliferative diabetic retinopathy without vitreous hemorrhage, PDR + VH, proliferative diabetic retinopathy with vitreous hemorrhage.

Because MANF is a secreted protein and certain amounts of vitreous proteins originate from the retina (Skeie and Mahajan, 2011), there is a significant possibility that MANF may be present in the vitreous. To determine whether MANF was present in the vitreous, the vitreous of human donors was extracted and used for Western blotting to measure the expression of MANF. We found that MANF was exactly present in human vitreous; specifically, a major protein band of the expected molecular weight, 25 kDa, was clearly identified (**Figure 8A**). The protein composition and content of the vitreous may change under physiological and pathological conditions of the retina. With the use of ELISA assay kits, we detected MANF in all vitreous fluid samples from different retinopathies, including proliferative diabetic retinopathy (with or without vitreous hemorrhage), macular holes, and retinal detachment (**Figure 8B**). The mean levels of MANF in the vitreous samples from patients with proliferative diabetic retinopathy (*n* = 21) (3.44 ± 1.07 ng/ml) and retinal detachments (*n* = 16) (3.43 ± 1.09 ng/ml) were significantly higher than those in patients with macular holes (*n* = 18) (1.21 ± 0.28 ng/ml) (*p* < 0.001). The MANF levels were clearly lower in the vitreous samples from patients with pure proliferative diabetic retinopathy (*n* = 8) (2.07 ± 0.14 ng/ml) compared with those from the patients with proliferative diabetic retinopathy with vitreous hemorrhage (*n* = 13) (3.77 ± 0.97 ng/ml) (*p* < 0.01).

## Discussion

There are three important findings in the current study: (1) MANF was detected in the vitreous humor and highly expressed in the RGCs. (2) Exogenous MANF protected the RGCs from hypoxia-induced cell injury and apoptosis both *in vitro* and *in vivo* by preventing ERS-mediated apoptosis. (3) MANF levels in the vitreous humor were significantly higher in patients with proliferative diabetic retinopathy and retinal detachments compared with patients with macular holes. This is the first study to investigate the neuroprotective effects of MANF on RGC function and apoptosis both *in vivo* and *in vitro*, and it is the first study to report that MANF is detectable in human vitreous humor and exhibits altered expression under pathological conditions.

Mesencephalic astrocyte-derived neurotrophic factor, with a molecular weight of 25 kDa, is widely expressed in mammalian tissues (Lindholm et al., 2008). Increasing evidence indicates that MANF promotes the survival of dopamine neurons at least partially by regulating the ER stress response, and MANF improves motor function more efficiently than other neurotrophic factors (Voutilainen et al., 2015). New research in the latest issue of *Science* suggests that MANF promotes tissue repair and successful regeneration in the retina (Neves et al., 2016). In view of these findings, we hypothesized that MANF may be expressed in the retina and protect the retinal tissue through particular mechanisms. To test this hypothesis, we needed to study the expression of MANF in the retinal tissue. Our results verified that MANF was highly expressed in RGCs, which provided a good foundation for our further studies.

An *in vitro* analysis of RGCs is a crucial and nearly indispensable tool for the study of retinal visual physiology and pathophysiology associated with various retinopathies and neuropathies, which cannot easily be realized in animal models. In addition, hypoxia is an important cause of many ocular diseases, including glaucoma (Li et al., 2011). Our *in vitro* study provided direct evidence of the protective effect of MANF on hypoxia-induced RGC loss. Moreover, CHOP, a key player in ER stress-mediated apoptosis (Oyadomari et al., 2002), and the pro-apoptotic protein cleaved caspase-3 were downregulated by MANF, which suggested that MANF exerts protective effects on RGCs and prevents ERS-mediated apoptosis.

The *in vitro* environment is relatively simple, and it was unknown whether MANF exerts a similar protective benefit on RGCs *in vivo*. To further examine the role of MANF in the survival of RGCs under pathological conditions, we used the COHT model, also known as ischemia-reperfusion, which has been widely used to investigate the mechanisms of RGC death (Sandalon et al., 2013). There is compelling evidence that RGC dysfunction occurs earlier and precedes the loss of RGC somas in glaucoma (Shou et al., 2003). Therefore, early functional protection is crucial for slowing the progression of glaucomatous optic neuropathy. The PhNR, which can be measured by a minimally invasive electrophysiological technique, has been reported to originate primarily from the RGCs and their axons (Porciatti, 2015). It provides a direct, objective assessment of the functional changes in the RGCs (Wilsey and Fortune, 2016). In the current study, a significant functional impairment in the RGCs was identified under COHT, while MANF treatment rescued the early PhNR deficit after 3 days of COHT, which indicated that MANF can protect against RGC functional damage in the early stage of COHT, even though RGC function was not returned to normal levels. However, with the progression of COHT, only a small trend of functional improvement was found. It is possible that with prolonged COHT, additional traumatic pathological pathways are activated such that MANF can no longer confer a sufficient protective effect. RGC apoptosis increased following the functional damage after IOP elevation. FG retrograde labeling showed that MANF effectively inhibited RGC apoptosis without decreasing IOP, and the early protection capacity was far greater than the later effects, which is consistent with the results of the functional assessment. Additionally, these results were further verified by quantitative analysis of survivin expression levels. Collectively, we can confirm that MANF plays a protective role in RGC function and survival under hypoxia both *in vitro* and *in vivo*.

Mesencephalic astrocyte-derived neurotrophic factor is a secreted protein and is detectable in human serum (Galli et al., 2016). Thus, we questioned whether MANF is detectable in the vitreous and whether vitreous MANF levels are associated with retinopathy. As we expected, MANF was found in the human vitreous, and MANF levels were elevated under pathological conditions, such as proliferative diabetic retinopathy and retinal detachment, compared with macular holes. The MANF expression levels were clearly lower in the vitreous samples from patients with pure proliferative diabetic retinopathy compared with those from patients with proliferative diabetic retinopathy with vitreous hemorrhage, and this finding may be due to the mixing of the vitreous with blood, as extremely concentrations of MANF are present in the serum (Galli et al., 2016). These data suggest that MANF in the human vitreous humor may be an interesting protein biomarker for the indirect detection of retinal disorders, which can provide indirect evidence for retinal pathology.

The limitations of this study include the small number of vitreous samples, which may not have been sufficient. A larger number of samples are needed for future studies. In addition, we used patients with macular holes as a healthy control group due to the lack of normal samples. Unfortunately, we also had no data on whether MANF expression levels are associated with the progression of retinal diseases or the expression of other cytokines in the vitreous. Finally, the origins and mechanisms of increased vitreous MANF have not yet been revealed.

## Conclusion

This study provides the first direct evidence to date that MANF is highly expressed in RGCs and that exogenous MANF can protect RGCs from hypoxia-induced cell injury and apoptosis in both a rat model of chronic glaucoma *in vivo* and hypoxia-induced RGC apoptosis *in vitro*. The mechanisms of MANF protection depended on the prevention of ERS-mediated apoptosis, not decreased IOP. Additionally, an interesting finding of potential clinical relevance in this study is that MANF protein can be detected in the vitreous. Furthermore, patients with proliferative diabetic retinopathy and retinal detachments showed a significantly higher MANF level compared with patients with macular holes. Thus, our results provide important evidence demonstrating that MANF may be a potential therapeutic protein for the treatment of a range of retinal pathologies either in the preclinical stage or after diagnosis to promote the survival of RGCs. Vitreous MANF may be a promising protein biomarker for the indirect assessment of retinal disorders, which could also provide indirect evidence of retinal pathology. Nevertheless, MANF requires further attention and investigation.

## Author Contributions

QW designs this work, revise it critically and finally approve the version to be published. J-HW contributed in the experimental design, revised the manuscript, collected and analyzed the data, T-TL and S-SD take part in some of the experimental studies, for example, western blotting, PCR analysis and cell culture. F-JG drafted, revised the manuscript and take part in a majority of the work. All authors read and approved the manuscript.

## Conflict of Interest Statement

The authors declare that the research was conducted in the absence of any commercial or financial relationships that could be construed as a potential conflict of interest.
